# The Mechanism of Volatile Oil of *Rhodiola tangutica* against Hypoxia-Induced Pulmonary Hypertension in Rats Based on RAS Pathway

**DOI:** 10.1155/2022/9650650

**Published:** 2022-09-06

**Authors:** Xingmei Nan, Zhanting Yang, Shanshan Su, Zhengnan Huang, Ke Ma, Shengrong Xu, E. Zhang, Dianxiang Lu, Zhanqiang Li

**Affiliations:** ^1^Medical College of Qinghai University, Xining 810001, China; ^2^Technical Center of Xining Customs, Key Laboratory of Food Safety Research in Qinghai Province, Xining, China; ^3^Officers College of PAP, Chengdu 610213, China; ^4^Research Center for High Altitude Medicine, Key Laboratory of High Altitude Medicine (Ministry of Education), Key Laboratory of Application and Foundation for High Altitude Medicine Research in Qinghai Province (Qinghai-Utah Joint Research Key Lab for High Altitude Medicine), Qinghai University, Xining, China

## Abstract

**Materials and Methods:**

Seventy-five male Sprague-Dawley (SD) rats were separated into control (Ctr), hypoxia (Hyp), and Hyp+VORA treatment (100 mg/kg/d, 80 mg/kg/d, and 40 mg/kg/d) groups in random. To achieve the chronic hypoxia condition, rats were kept inside the hypobaric chamber with automatically adjusted inner pressure as well as oxygen content equal to those of 4500 m in altitude for 4 continuous weeks. After 4 weeks, the rats' physiological parameters were determined (mean pulmonary artery pressure (mPAP); right ventricular hypertrophy index (RVHI)). Based on hematoxylin and eosin (HE) staining and transmission electron microscope (TEM), morphological features of their lung tissues were also analyzed. Proliferation of pulmonary arterial smooth muscle cells (PASMCs) was detected by MTS Cell Proliferation Colorimetric assay. The levels of glutathione (GSH), malondialdehyde (MDA), and superoxide dismutase (SOD) in PASMCs were detected through corresponding kits, respectively. The protein levels in PASMCs and HPH rats were evaluated by Western blot (WB). Chemical components of VORA were detected through gas chromatography-mass spectrometer (GC-MS).

**Results:**

After induced by hypoxia for 4 weeks, the mPAP and RVHI levels were increased significantly in hypoxia group in contrast to the Ctr group, indicating the establishment of HPH rat model. The subsequent administration of VORA decreased the mPAP and RVHI level. The vascular wall thickness and lumen size were also decreased after treated by VORA compared with Hyp group. Meanwhile, VORA suppressed the proliferation and oxidant stress in PASMCs. Therefore, the effect of VORA on decreasing vascular wall thickening and lumen size could be related to its antiproliferation effect on PASMCs. In addition, compared to the Hyp group, VORA downregulated the ACE, AngII, and AT1R protein expressions but increased ACE2 and MAS protein expressions (*P* < 0.05). A total of 48 constituents in VORA were identified by GC-MS in comparison with reference standards as well as the reference pieces of literatures.

**Conclusions:**

HPH rat model as established based on the significant increased mPAP and RVHI. VORA presented a significant antihypoxia function plus an inhibiting effect on PASMC proliferation induced by hypoxia. Moreover, VORA treatment inhibited oxidative stress among PASMCs. With regard to the mechanism, VORA reduced ACE, AngII, and AT1R protein expressions but increased ACE2 and MAS protein expressions. There were 48 constituents in VORA identified by GC-MS.

## 1. Introduction

As a result of the pulmonary vasculature remodeling, hypoxia-induced pulmonary hypertension (HPH) has been characterized by elevated pulmonary vascular resistance as well as increased pulmonary pressure [1], which eventually resulted in right heart failure and deaths in patients [2]. The vasoconstriction and the pulmonary vessel wall remodeling have been playing a role in increasing pulmonary vascular resistance in HPH [3]. One of HPH's mechanisms at present is related to the secretion of vasoactive components from vascular endothelial cells and vascular smooth muscle cells with the capacity of regulating the contraction and proliferation of vascular smooth muscle in a paracrine manner and an autocrine manner [4]. These vasoactive components are dominated by key factors in the renin-angiotensin system (RAS) pathway, including angiotensin-converting enzyme (ACE)/angiotensin (Ang) II/Ang II type 1 receptors (AT1R) as well as ACE2/Ang-(1-7)/Mas receptor ([5]).

As a traditional Tibetan medicine commonly used for high-altitude sickness, the development and utilization of *Rhodiola tangutica* (*Maxim.*) S.H. Fu (*Rhodiola tangutica*) should not only effectively protect the plant resources but also pay attention to the efficient utilization of natural resources by using modern high and new technology and change the traditional single way of resource utilization. Study the active chemical structure and pharmacological mechanism of natural products, make value-added utilization of natural resources in multiple ways, and improve the actual utilization rate of resources. Previous study showed that *Rhodiola tangutica* observably downregulated the index of right ventricular hypertrophy (RVH) and the mean pulmonary artery pressure (mPAP), as well as thickness of pulmonary arteriole's wall, and also caused pulmonary capillary vascular remodeling, as previously reported. Volatile oil in *Rhodiola tangutica* (*Maxim.*) S.H. Fu (VORA) is an important chemical composition of *Rhodiola tangutica*. The chemical compositions of VORA, instead of its pharmacological actions, have attracted a lot of attention. Therefore, this study was designed to explore whether VORA could alleviate HPH in rats and its underlying mechanism.

## 2. Materials and Methods

### 2.1. Materials and Reagents

*Rhodiola tangutica* was purchased from Tibetan Traditional Medical Hospital of Zhiduo County, which is recognized as the authority in the field of Tibetan medicine, in Yushu Tibetan Autonomous Prefecture of Qinghai Province. Professor Dejun Zhang of the College of Eco-Environmental Engineering in Qinghai University ascertained the raw plant materials used in this study. The specimen (No. 2015-12) was manufactured by the pharmacy department. Anti-ACE antibodies (ab29), anti-AngII antibodies (ab199728), anti-AT1R antibodies (ab134175), anti-ACE2 antibodies (ab193379), anti-MAS antibodies (ab193379), and anti-*β*-actin antibodies (ab8226) were purchased from Abcam Biotechnology, USA. The fetal bovine serum (FBS, Thermo Fisher Scientific, USA), DMEM, FBS (Gibco), penicillin (100 U/mL), and streptomycin (100 U/mL) were used together with MTS Cell Proliferation Colorimetric assay (Sigma, USA); BCA protein assay kit (Beyotime, Shanghai, China); and glutathione (GSH), malondialdehyde (MDA), and superoxide dismutase (SOD) kit (Nanjing Jiancheng, Nanjing, China).

### 2.2. Preparation for Extraction

One kilogram of raw *Rhodiola tangutica* was powdered and soaked for 8 h. VORA was extracted through continuous heated and refluxed steam until to no oil dropped down any more (yield 3.2%, *w*/*w*). Then, VORA was dried with anhydrous Na_2_SO_4_ and preserved at -4°C for further research. The dose of 40 mg/kg/day, 80 mg/kg/day, and 100 mg/kg/day of VORA suspended in distilled water (contained 0.01% Tween-80) was used in animal experiment for 4 weeks by gavage administration.

### 2.3. Animals

Seventy-five Sprague-Dawley (SD) rats (weighted 135-160 g, male) were provided by Animal Center of Xi'an Jiaotong University, China. Throughout the experiments, rats were allowed *ad libitum* feeding on water as well as standard pelleted diet (temperature: 22 ± 2°C, relative humidity: 45–55%). The experimental rats were categorized into 3 groups randomly: the control (Ctr), hypoxia (Hyp), and Hyp+VORA (100 mg/kg/d, 80 mg/kg/d, and 40 mg/kg/d) groups. Rats in Hyp group were kept in the hypobaric chamber with the pressure as well as oxygen content equal to those of 4500 m in altitude for 4 continuous weeks. Every possible effort was tried with the aim to reduce the animal's distress and death in addition to limiting the number of experimental rats. This experimental protocol was approved by the Institutional Animal Care and Use Committee of Qinghai University in consistence with the animal management rules of the Chinese Ministry of Health.

### 2.4. Hemodynamic and Histopathological Staining

Rats were urethane-anesthetized (1.0 g/kg, i.p.) after 4-week experiment, with their weight recorded. The mPAP was detected through right cardiac catheterization. Right ventricle (RV), left ventricle (LV), and interventricular septum (S) of each rat were separated based on the ventricular septal edge. The weight was measured for each rat and was used to determine the right ventricle index (RV/LV+S, RVHI). Then, the left lung tissue was collected, followed by the fixation in paraform (4% [*w*/*v*]). Subsequently, paraffin sections (5 *μ*m) were made. Morphometric analysis was carried out based on hematoxylin and eosin (HE) staining. The thickness of distal pulmonary arteriole's wall was checked through light microscope.

### 2.5. Cell Culture

After being anesthetized, the pulmonary arteries of those healthy rats kept under normoxia were rapidly dissected. The pulmonary artery smooth muscle cells (PASMCs) were cultured in vitro as the method reported by Skalli [6]. Adherent cells were trypsinized at 70-80% confluence with a split ratio of 1 : 3. The cells are recultured in DMEM containing FBS (5% (*v*/*v*), Gibco), penicillin (100 U/mL), and streptomycin (100 U/mL) at the temperature of 37°C. Finally, cells of the 4th to 6th generations were adopted for the following experiments.

### 2.6. Assays for Cell Proliferation

MTS assay was adopted to detect the proliferation of PASMCs by formazan absorbance. After seeded in 96-well plates (10,000 cells/cm^2^), PASMCs were divided into the Ctr group (cells were cultured under normoxia (N_2_[74%], CO_2_[5%], and O_2_[21%]), Hyp group (cells were cultured under hypoxia condition, (N_2_ [93%], CO_2_[5%], and O_2_[2%]), and Hyp+VORA group (cells were cultured under hypoxia condition and intervened with different concentrations of VORA [0, 0.5, 0.6, 0.7, 0.8, 0.9, and 1.0 mg/mL]). Cells in each group were incubated for 10 h with serum free DMEM for cell synchronization after cell adhesion. Then, cells were treated with VORA (0, 0.5, 0.6, 0.7, 0.8, 0.9, and 1.0 mg/mL) for 12 h and 24 h in normoxia or hypoxia condition, which was named as N12 h, N24 h and H12 h, H24 h groups, respectively. Comparatively, plain DMEM was adopted as the negative control. Subsequently, the cells were treated with 10 *μ*L MTS (Sigma, USA) for another 3 h at 37°C. Then, the absorbance was measured at 490 nm.

### 2.7. Measurement of Oxidative Stress

The parameters for oxidative stress in PASMCs were evaluated complying with the instructions provided by the manufacturer (Solarbio Life Sciences Co., Ltd., Beijing, China), including the MDA content, SOD, and GSH-Px activity, which were measured at 532 nm, 550 nm, and 412 nm, respectively.

### 2.8. Western Blotting (WB) Analysis

Protein expression levels of ACE, AngII, AT1R, ACE2, and MAS in lung tissue and PASMCs were studied through WB. The lung tissue and the cells were collected and frozen and then homogenized in RIPA buffer, followed by the centrifugation (10,000*g*, 15 minutes, and 4°C). Subsequently, the concentrations of the proteins were measured using BCA protein assay kit, and the result was compared with those in the conventional sample of bovine serum albumin (BSA). After being segregated through SDS-PAGE, fifty micrograms of proteins per lane was transferred to polyvinyl difluoride (PVDF) membranes, which were further blocked with Tris-buffered saline with Tween-20 (TBST) containing nonfat dry milk (5%) and incubated with anti-ACE antibodies, anti-AngII antibodies, anti-AT1R antibodies, anti-ACE2 antibodies, anti-MAS antibodies, and anti-*β*-Actin antibodies at a concentration of 1: 5000 (ACE, AngII, AT1R, and ACE2), 1 : 10000 (MAS), for the whole night at 4°C, followed by the incubation with goat anti-mouse/anti-rabbit immunoglobulin G at a concentration 1 : 5000. The enhanced chemiluminescence (ECL) kit (Biyuntian Biotech Institute, Beijing, China) was used for visualization. Equal lane loading was assessed using *β*-actin.

### 2.9. GC-MS Analysis

The injector temperature was 250°C. The program of GC oven was set as follows: the injection volume was 1.0 *μ*L, with no shunt injection. After maintaining the initial temperature at 50°C for 2 min, the temperature was increased at a rate of 2°C/min to 120°C (held for 5 min), and further increased at a rate of 2°C/min to 190°C (held for 2 min), and finally increased at a rate of 20°C/min to 280°C (held for 2 min). Helium was used as the carrier gas at a flow rate of 1.2 mL/min through the column. The MS conditions were as follows: temperature of ion transport tube, 250°C; electronic impact ion source, 280°C; ionization energy, 70 eV; and mass range, 50–600.

### 2.10. Statistical Analysis

The results were described as mean ± standard deviation (SD). Statistical analysis was performed by one-way analysis of variance (ANOVA). Statistical significance was defined as *P* ≤ 0.05.

## 3. Results

### 3.1. Effect of VORA in HPH Rats

#### 3.1.1. The Effect of VORA on mPAP in HPH Rats Was Explored Primarily

**Figure 1:**
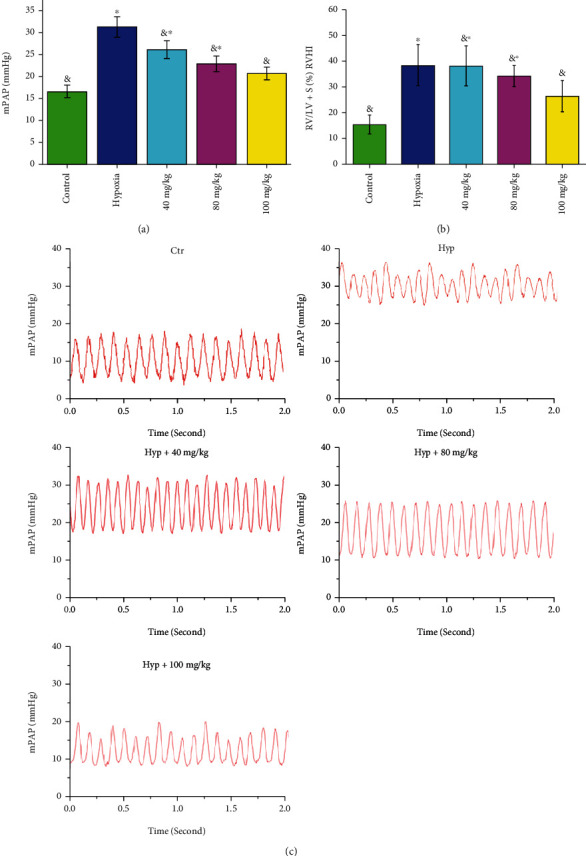
Effect of volatile oil of *Rhodiola tangutica* (VORA) on HPH rats. There were three groups, including the control (Ctr) group, hypoxia (Hyp) group, and Hyp+VORA group. (a) Mean pulmonary arterial pressure (mPAP), (b) Right ventricular hypertrophy index (RVHI), and (c) mPAP waves for groups. Data was presented as means ± standard deviation (SD) (^&^*P* < 0.05 vs. Ctr group, ^∗^*P* < 0.05 vs. Hyp group, *n* = 8).

The result showed that compared to the normal group, the levels of mPAP (*P* < 0.05, Figures 1(a) and 1(c)) and RVHI (*P* < 0.05, Figure 1(b)) were significantly increased in the model group, indicating the establishment of HPH rat model. After being treated by VORA, mPAP and RVHI were decreased in HPH rats (*P* < 0.05, Figures 1(a)–1(c)).

#### 3.1.2. HE Staining Showed that the Endothelial Cells Located within the Arteriole's Wall Were Evenly Distributed in Ctr Group

**Figure 2:**
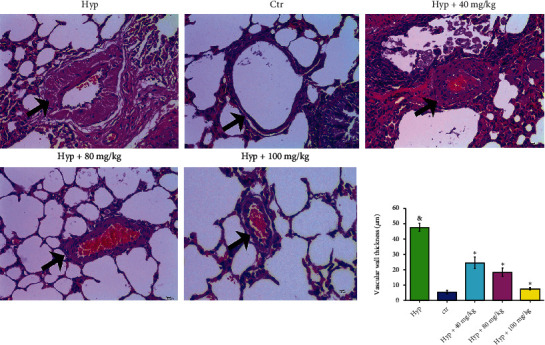
Effect of VORA on arteriole's wall thickness as well as lumen size of right ventricular tissues among HPH rats using HE staining (400x, *n* = 4). Vascular changes were shown by the black arrows.

By contrast, the arteriole's wall was thickened and lumen diameter was decreased with the proliferation of PASMCs in HPH group. In Hyp+VORA group, the lung tissues exhibited decreased thickness of arteriole's wall and lumen size compared with HPH rats (Figure 2).

#### 3.1.3. Effect of VORA on mRNA and Protein Expression Levels of ACE, AngII, AT1R, ACE2, and MAS in HPH Rats Was Detected by Western Blotting and qRT-PCR

**Figure 3:**
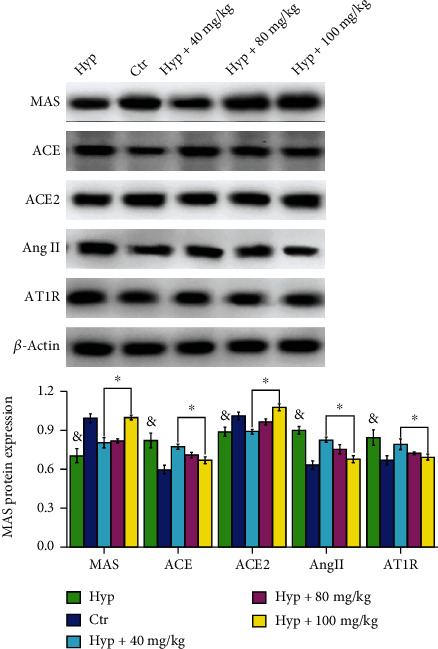
Effects of VORA on protein expression levels of ACE, AngII, AT1R, ACE2, and MAS among HPH rats. Data was presented as means ± standard deviation (SD) (^&^*P* < 0.05 vs. Ctr group, ^∗^*P* < 0.05 vs. Hyp group, *n* = 3).

The results showed that compared with the Hyp group, the mRNA and protein levels of either ACE2 or MAS were significantly increased by VORA (*P* < 0.05, Figure 3); by contrast, the levels of ACE, Ang2, and AT1R were decreased by VORA (*P* < 0.05, Figure 3).

### 3.2. Effect of VORA on PASMCs

#### 3.2.1. MTS Assay Was Carried Out to Assess the Impact of VORA to the Hypoxia-Induced Proliferation in PASMCs

**Figure 4:**
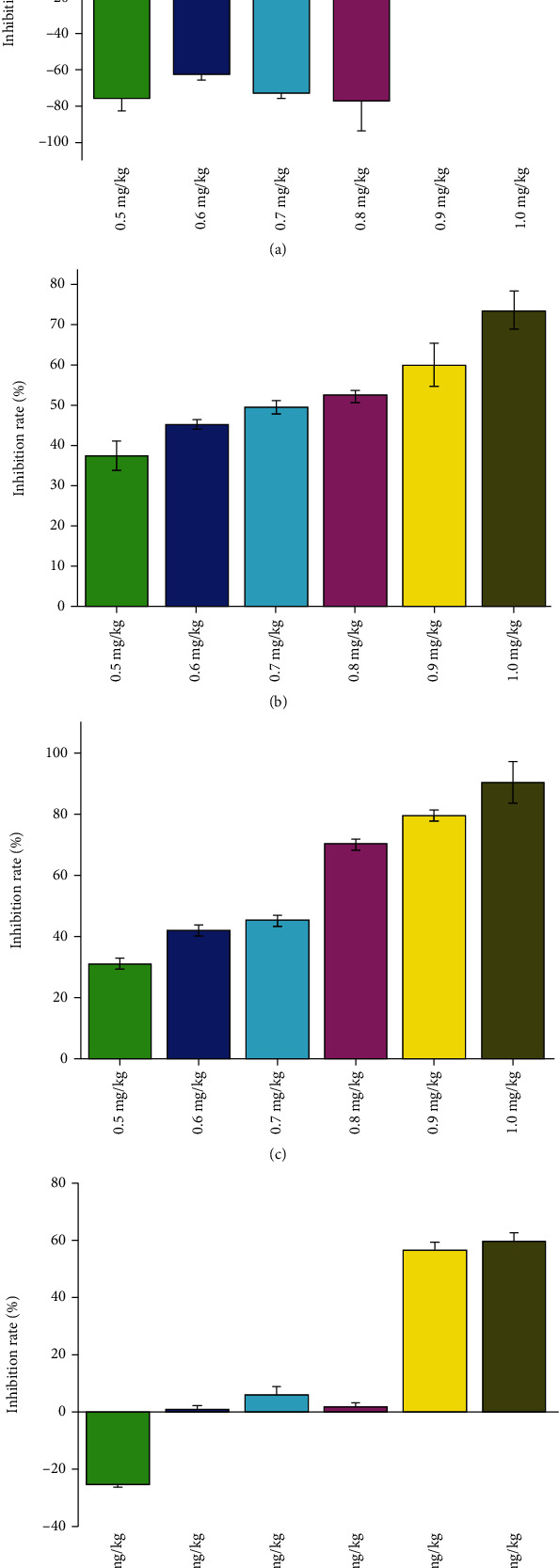
Effect of VORA in PASMC's proliferation. (a) indicated the inhibiting rates of VORA on PASMCs under normal condition for 12 h, (c) showed the inhibiting rates of VORA on PASMCs under hypoxia condition for 12 h, (b) indicated the inhibiting rates of VORA on PASMCs under normal condition for 24 h, and (d) showed the inhibiting rates of VORA on PASMCs under hypoxia condition for 24 h. Data was presented as means ± standard deviation (SD).

After the 12 and 24 h exposure under normoxia or hypoxia, PASMCs were incubated with VORA (0.5, 0.6, 0.7, 0.8, 0.9, and 1.0 mg/mL), the same as previous regimen. Results showed that hypoxia significantly stimulated PASMC's proliferation; however, VORA treatment inhibited the proliferation in a concentration-dependent manner both in 12 and 24 h exposures (Figure 4, *P* < 0.05). VORA (0.5, 0.6, 0.7, and 0.8 mg/mL) showed no significant inhibition for the cells under normoxic condition (Figure 4). Based on these results, it can be inferred that VORA inhibited hypoxia-induced proliferation of PASMCs effectively. Nevertheless, VORA showed lower cytotoxicity in normoxia condition in 12-hour exposure at the concentration of 0.5-0.8 mg/mL. Therefore, 12 h was selected as the main observation time point for VORA treatment. At the same time, take values of IC_50_ = 0.8 mg/mL up and down in equal difference columns (Figure 4).

#### 3.2.2. In Oxidant Stress Study

**Figure 5:**
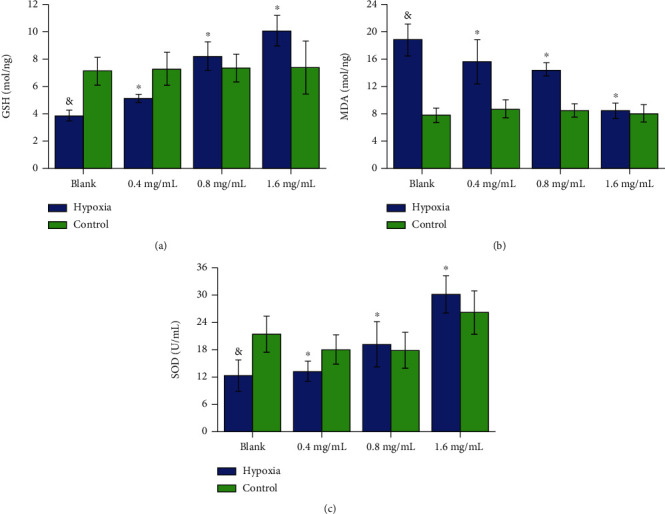
Role of VORA in oxidant stress of PASMCs. (a) and (b) indicated the content of GSH and MDA in PASMCs and (c) showed the SOD activity in PASMCs. Data was presented as means ± standard deviation (SD) (^&^*P* < 0.05 vs. Ctr group, ^∗^*P* < 0.05 vs. Hyp group, *n* = 4).

Our results showed that in Hyp group, the content of MDA was increased, while SOD and GSH activities were decreased in PASMCs. After being treated by VORA, MDA content was significantly decreased, but SOD and GSH activities were increased by contrast (*P* < 0.05, Figure 5).

#### 3.2.3. The mRNA and Proteins in PASMCs Were Extracted and Detected by qRT-PCR and WB

**Figure 6:**
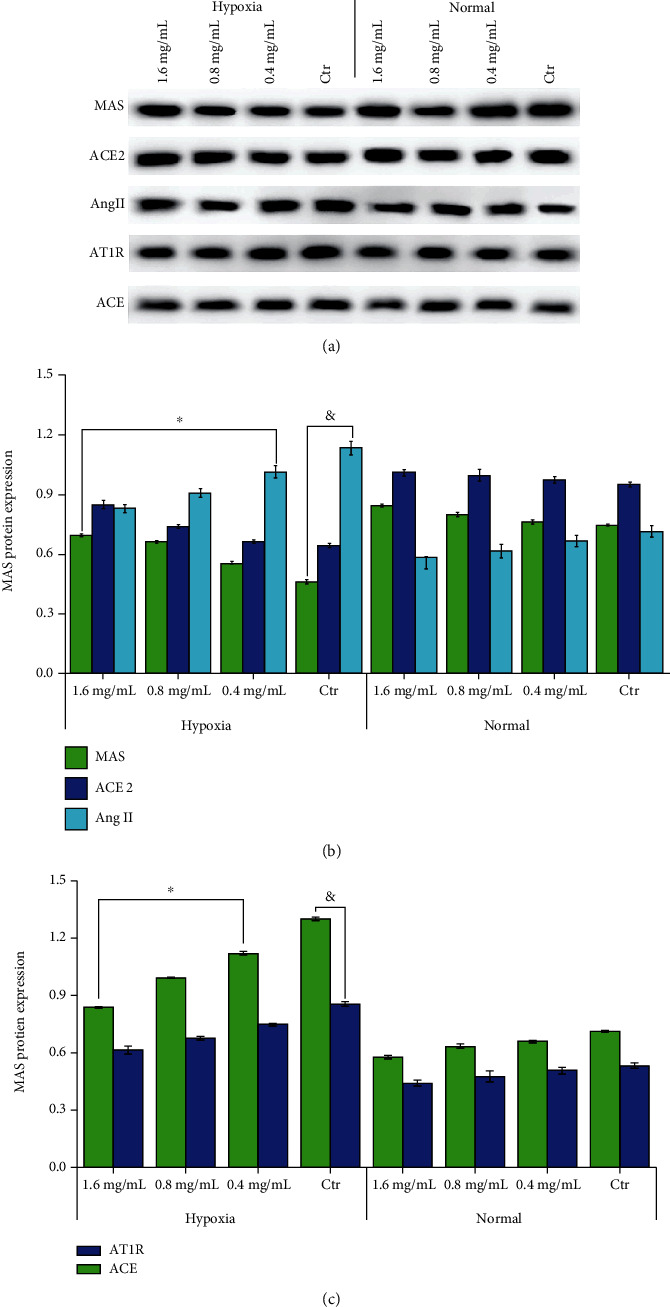
Effects of VORA on ACE, AngII, AT1R, ACE2, and MAS protein expression levels in PASMCs. Data was presented as means ± standard deviation (SD) (^&^*P* < 0.05 vs. Ctr group, ^∗^*P* < 0.05 vs. Hyp group, *n* = 3).

According to our results, compared to the Ctr group, ACE, AngII, and AT1R protein expressions were increased but ACE2 and MAS protein expressions were decreased in Hyp group with statistical significance (Figure 6, *P* < 0.05). After being treated by VORA, the levels of ACE, AngII, and AT1R were decreased while ACE2 and MAS levels were increased compared with Hyp group (Figure 6, *P* < 0.05).

### 3.3. GC-MS Analysis

**Figure 7:**
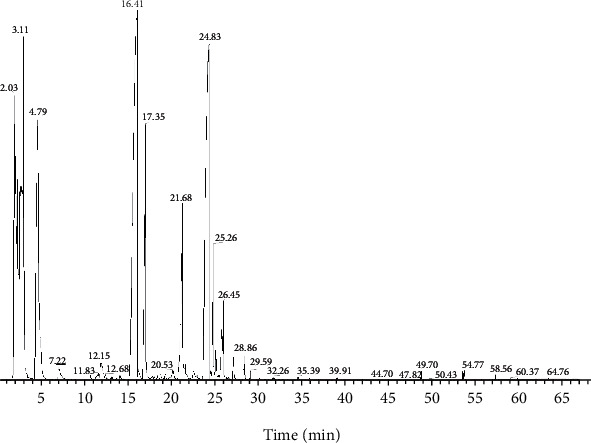
Chromatogram of VORA in GC-MS.

As shown in Figure 7, the total ion flow diagram (Figure 7) was obtained by GC-MS, and its effective components were analyzed and identified (Table 1). From the results, we found that the chemical constituents of volatile oil from *Rhodiola tangutica* are complex, mainly consisted by terpenoids, alcohols, esters, alkane, and so on. The compounds accounting for >2% included 3-methyl-2-buten-1-ol (14.4%), linalool (14.01%), geraniol (18.42%), (-)-myrtenol (9.63%), 4 (1-methylethenyl)-1-cyclohexe-ne-1-methanol (4.16%), P-cymen-7-ol (2.46%), 1-octanol (20.19%), and 1-decanol (7.2%). The relative content is 90.2% (Figure 7).

## 4. Discussion

So far, it has been found that hypoxia can compensate for the increase of lung ventilation and contraction of pulmonary blood vessels [7]. Persistent hypoxia will directly induce the remodeling of pulmonary blood vessels, which will lead to HPH development, even heart failure. In modern studies, the mechanism of pulmonary vascular remodeling induced by hypoxia is focused on the secretion of vasoactive components by vascular endothelial cells, including the regulations of the relaxation, contraction, and proliferation of vascular smooth muscle in a paracrine way [8]. Meanwhile, vascular smooth muscle cells can also produce many vasoactive components in an autocrine way, which are the key factors in renin-angiotensin system pathway.

Aromatic substances in traditional Chinese medicine have been used as the main effective substances. As a unique active component of *Rhodiola tangutica*, VORA's medical value has not been paid enough attention. As previously reported, the bioactive fraction of *Rhodiola tangutica* could significantly reverse the hypoxia-induced RVH, pulmonary artery wall thickness, and ultrastructure injury [9] as well as the changes in hematological indexes among rats with HPH. In this study, we also detected the effect of VORA in HPH rats, and it showed that VORA treatment could significantly reverse the abnormal proliferation of PASMCs induced by hypoxia [9].

RAS is a complex cardiovascular regulatory system, which is a signal pathway to regulate the homeostasis of vascular functions [10]. In the past 40 years, it has been clear that RAS exists in most tissues, including the lung. RAS is mainly composed of two antagonistic and coordinated axes (ACE-Ang II-AT1R and ACE2/Ang-(1-7)/Mas) [11]. ACE could enhance the contraction of blood vessels and promote the proliferation of vascular smooth muscle and endothelial cells, playing an important role in the occurrence and development of pulmonary hypertension [11]. Besides, the activity of ACE directly determined the formation of Ang Ⅱ and the binding with AT1R, producing strong vasoconstriction. ACE is mainly distributed in vascular endothelial cells, while ACE2 is widely distributed in the heart. It was found that ACE2 could hydrolyze Ang II to Ang (1 ≤ 7), which has the capacity of inhibiting the proliferation of PASMCs [12]. Therefore, as an important vascular protector in RAS system, ACE2 protected blood vessels and downregulated inflammatory factors by inhibiting the vasoconstriction, cell proliferation, and fibrosis [12]. The deletion of MAS receptor could interrupt the balance between its oxidant and antioxidant effects, contributing to vascular endothelial dysfunction and eventually leading to pulmonary hypertension [13, 14]. It was found that the balance between ACE-Ang II-AT1R axis and ACE2-Ang (1 ≤ 7)-MAS axis was damaged in HPH, which may be potential as its new treating target [15]. In this research, the impact of VORA to the protein expressions of ACE, Ang II, and AT1R in HPH rats and PASMCs were investigated. WB analysis showed that VORA could significantly reduce the protein expressions of ACE, Ang II, and AT1R in lung tissue in HPH rats and PASMCs (*P* < 0.05). The protein levels of ACE2 as well as MAS were significantly decreased after induced by hypoxia and then increased by VORA treatment in HPH rats and PASMCs (*P* < 0.05).

*Rhodiola tangutica* is an essential herbal of traditional Tibetan medicine. Volatile oil is an effective component with multiple biological activities. In order to reveal the chemical constituents of VORA against HPH, 48 compounds were identified by GC-MS method. Flavonoids and phenylalanine in VORA have been considered to be effective substances against hypoxia [16]. Unexpectedly, VORA also has a good antihypoxia effect. Furthermore, we also found that VORA can significantly inhibit the abnormal proliferation of hypoxia-stimulated PASMCs.

## 5. Conclusions

This study investigated the intervention mechanism of volatile oil of *Rhodiola tangutica* (VORA) in HPH. 48 chemical components were detected in VORA using the GC-MS method. Our study suggested that it may be a potential HPH-preventive agent. We found that the administration of VORA reduced the mPAP in HPH rats based on the improvement of pulmonary small vessel morphology and the reversal of PASMC proliferation induced by hypoxia whose mechanism was related to the decreased ACE, Ang II, and AT1R expression levels, as well as the increased ACE2 and MAS levels. Based on the above facts, VORA has the potential to treat HPH. Further research on elucidating the material base and mechanism in detail is ongoing.

## Figures and Tables

**Table 1:** Chemical composition of VORA.

No.	*t_R_*/min	Identification	Molecular	Relative content (%)
1	4.79	3-Methyl-2-buten-1-ol	C_5_H_10_O	14.4
2	7.22	1-Aminocyclopropanecarboxylic acid	C_4_H_7_NO_2_	0.67
3	10.92	2,6,6-Trimethyl-2-vinyltetrahydro-2H-pyran	C_10_H_18_O	0.25
4	16.41	1-Octanol	C_8_H_18_O	20.19
5	17.35	Linalool	C_10_H_18_O	14.01
6	19.11	(S)-6-Methyl-1-octanol	C_9_H_20_O	0.24
7	20.52	Terpinen-4-ol	C_10_H_18_O	0.41
8	21.68	(-)-Myrtenol	C_10_H_16_O	9.63
9	23.35	Tridecanoic acid, methyl ester	C_11_H_16_	0.19
10	24.83	Geraniol	C_10_H_18_O	18.42
11	25.26	1-Decanol	C_10_H_20_O	7.2
12	25.56	6,6-Dimethyl-bicyclo[3.1.1]hept-2-ene-2-methanol	C_10_H_16_O	1.37
13	26.25	P-cymen-7-ol	C_10_H_14_O	2.46
14	26.45	4-(1-Methylethenyl)-1-cyclohexene-1-methanol	C_10_H_16_O	4.16
15	27.62	(4-Propan-2-ylcyclohexa-1,4-dien-1-yl)-methanol	C_10_H_16_O	1.21
16	28.86	(4-Isopropyl-1,3-cyclohexadien-1-yl)-methanol	C_10_H_16_O	1.33
17	29.59	Geranyl acetate	C_12_H_20_O_2_	0.48
18	30.67	Methyleugenol	C_11_H_14_O_2_	0.08
19	30.31	1-Butoxy-2-methyl-2-butene,(*E*)-	C_9_H_18_O	0.02
20	31.76	P-Mentha-1,8-dien-7yl-acetate	C_12_H_18_O_2_	0.03
21	32.26	4-(2,6,6-Trimethyl-cyclohex-1-enyl)-butan-2-ol	C_13_H_24_O	0.16
22	32.45	5,9-Undercadien-2-one, 6,10-dimethyl,(*Z*)-	C_13_H_22_O	0.07
23	32.63	6,10-Dimethylundeca-5,9-dien-2-ol	C_13_H_24_O	0.04
24	32.95	3,7-Dimethyl-6-octadien-1-ol	C_10_H_18_O	0.08
25	33.42	1-Undecanol	C_11_H_24_O	0.05
26	35.39	1,3-Benzodioxole,4-methoxy-6-(2-propenyl)-	C_11_H_12_O_3_	0.15
27	35.67	3,5,9-Undecatrien-2-one, 6,10-dimethyl-	C_13_H_20_O	0.02
28	36.69	3-(3,4,5-Trimethoxyphenyl)-1-propene	C_12_H_16_O_3_	0.11
29	37.56	Hexanoic acid, octyl ester	C_14_H_28_O_2_	0.05
30	39.91	Farnesol	C_15_H_26_O	0.15
31	42.83	Methyl tetradecanoate	C_15_H_30_O	0.04
32	43.07	7-Methoxymethyl-2,7-dimethyl cyclohepta-1,3,5-triene	C_11_H_16_O	0.02
33	43.85	Geranyl isobutyrate	C_14_H_24_O_2_	0.04
34	44.70	Octanoic acid, octyl ester	C_16_H_32_O_2_	0.05
35	47.00	2-Pentadecanone,6,10,14-trimethyl-	C_18_H_36_O	0.01
36	47.82	Phthalic acid diisobutyl ester	C_16_H_22_O_4_	0.03
37	49.43	6,10,14-Trimethyl-5,9,13-pentadecatrien-2-one	C_18_H_30_O	0.02
38	49.70	Hexadecanic acid, methyl ester	C_17_H_34_O_2_	0.5
39	50.43	*cis*-3,7-Dimethyl-2,6-octadien-1-ol, propionate	C_13_H_22_O_2_	0.03
40	50.91	Dibutyl phthalate	C_16_H_22_O_4_	
41	54.77	9,12-Octadecadienoic acid, methyl ester	C_19_H_34_O_2_	0.68
42	54.92	(*Z*,*Z*,*Z*)-9,12,15-Octadecatrienoic acid, methyl ester	C_19_H_32_O	0.51
43	55.52	Tridecanoic acid methyl	C_14_H_28_O_2_	0.02
44	56.93	Hexadecane	C_16_H_34_	0.03
45	58.65	N-Heneicosane	C_21_H_44_	0.26
46	60.37	Tetrade canal	C_14_H_28_O	0.06
47	60.93	Oleic acid	C_18_H_34_O_2_	
48	61.18	Tetracosane	C_24_H_25_	0.05

TR: retention time.

## Abbreviations

VORA: Volatile oil of *Rhodiola tangutica*
HPH: Hypoxia-induced pulmonary hypertension
mPAP: Mean pulmonary artery pressure
RVHI: Right ventricular hypertrophy index
HE: Hematoxylin and eosin staining
GC-MS: Gas chromatography-mass spectrometer
RAS: Renin-angiotensin system pathway
MAS: receptor MAS
ACE: Angiotensin-converting enzyme
Ang: Angiotensin
Ang II: Angiotensin II
AT1R: Ang II type 1 receptors
ACE2: Angiotensin-converting enzyme2
GSH: Glutathione
MDA: Malondialdehyde
SOD: Superoxide dismutase
FBS: Fetal bovine serum
PASMCs: Pulmonary arterial smooth muscle cells
SPF: Specific pathogen free
SD rat: Sprague-Dawley rat.
